# Translation of a gestational diabetes nutrition model of care into practice: results from an implementation project

**DOI:** 10.1186/1472-6963-14-S2-P139

**Published:** 2014-07-07

**Authors:** Shelley Wilkinson, Sally McCray, Michael Beckmann, H David McIntyre

**Affiliations:** 1 Mater Research Institute, University of Queensland, Brisbane, Australia; 2 Mater Mothers’ Hospitals’, Brisbane, Australia; 3 Mater Health Services, Department of Nutrition & Dietetics, University of Queensland, Brisbane, Australia

## Background

Reduced need for insulin therapy in Gestational Diabetes Mellitus (GDM) has been documented in a study validating American Nutrition Practice Guidelines, which recommend at least 3 dietitian visits. No Australian GDM Nutrition Practice Guidelines exist and systematic delivery of dietetic care to women with GDM does not occur in Australia. This paper evaluates a theory-informed implementation plan to translate a dietetic model of care based on the American guidelines in an Australian maternity hospital.

## Materials and methods

The planned implementation consisted of a 9-month pre (usual care)/post (new model of care) design with a month for ‘integration’ across 2012-2013. Primary outcomes were uptake of the new dietetic schedule (process) and requirement for pharmacotherapy (clinical). Secondary outcomes were change in: staff’s awareness, knowledge, and acceptance of the model, and change in client satisfaction (process), and in dietary indices, physical activity levels, and key maternal and infant outcomes (clinical).

## Results

Both phases only ran for 7 months; integration required 4 months. Pre-intervention, only one of the 91 women with GDM seen received ≥ 1 dietetic follow-up appointment. Post-intervention, significantly more women (50.6%) received best-practice care (2+reviews) (p = 0.02). However, due to heavy clinical demand, only 31.5 % of the 162 women seen after the change in practice received best-practice individual dietitian review at their first visit. Clinically-relevant trends were seen in changes in medication requirements; the percentage of women requiring pharmacologic treatment decreased from 31.1% to 26.9%. This was more pronounced in women who received best-practice care (25.0% (yes) vs. 27.2% (no)).

Only a small change in glycemic index of women’s diet occurred after seeing a dietitian, pre- to post- implementation (-2.0 ± 4.4 vs. -3.0 ± 5.0). However, this was significant between women who received best practice care (-7.9 ± 6.1) and not best practice care (-2.1 ± 4.4, p = 0.014), and also pre-intervention women (p=0.01). Differences in physical activity levels were clinically, but not significantly different (15.5 mins vs 9l.8mins/week). Client satisfaction remained high over the project, between 4.3-4.7/5. Staff are currently being surveyed regarding their guideline knowledge and acceptance. Clinical outcomes are currently being examined.

## Conclusions

This implementation project was successful in increasing the proportion of women seen according to best practice. Service limitations impaired the delivery of optimal care. Partial adherence to the model of care may have attenuated changes in medication requirements and dietary patterns. Full adherence may have resulted in even greater changes.

